# Supplementary material for: New Emerging Therapies Targeting PI3K/AKT/mTOR/PTEN Pathway in Hormonal Receptor-Positive and HER2-Negative Breast Cancer—Current State and Molecular Pathology Perspective
Source: Cancers (Basel). 2024 Dec 24;17(1):16. doi: 10.3390/cancers17010016 (PMC11718791; doi:10.3390/cancers17010016)
Supplement: Supplementary file 1 [file cancers-17-00016-s001.zip › cancers-3333162-supplementary.pdf]

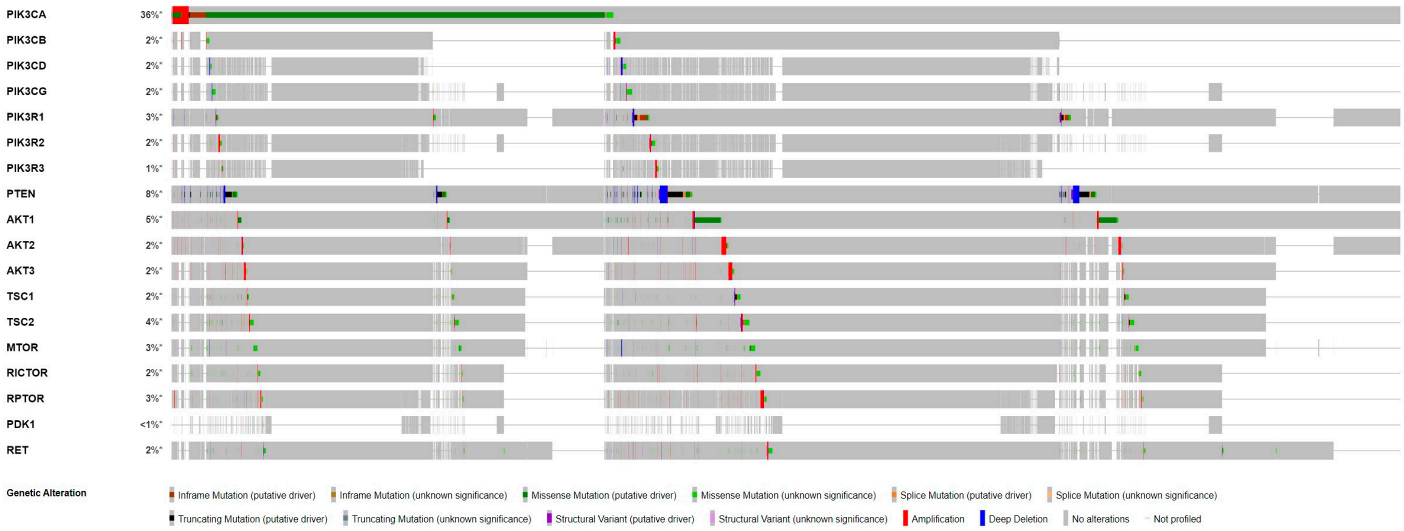

Supplemental Figure S1. Genomic alterations in selected PI3K pathway genes in breast cancer from cbioportal GENIE cohort.
